# Enhancing melatonin biosynthesis in crops through synthetic genetic circuits: A strategy for nutritional fortification in soybean and stress resistance in cotton

**DOI:** 10.1111/pbi.70253

**Published:** 2025-07-07

**Authors:** Yue Shi, Zegang Han, Wanying Zhang, Lu He, Zhuolin Shi, Xiaowei Ma, Ji Zhou, Zhanfeng Si, Yan Hu, Tianzhen Zhang

**Affiliations:** ^1^ Zhejiang Provincial Key Laboratory of Crop Genetic Resources, The Advanced Seed Institute, Plant Precision Breeding Academy College of Agriculture and Biotechnology, Zhejiang University Hangzhou China

**Keywords:** melatonin, synthetic genetic circuits, BUFFER gates, dietary supplements, biofortified crops

## Abstract

Melatonin has gained considerable prominence in the treatment of insomnia that significantly impacts one‐third of the global population. The production of melatonin remains challenging due to limitations in current methods. Thus, there is an urgent need for developing more efficient and innovative production techniques. Here, we demonstrated the potential of crop seeds as a platform for melatonin synthesis by engineering multiple BUFFER genetic circuits using synthesized transcriptional regulators, which enhance expression precision, orthogonality and thresholds. Biofortified soybeans exhibited a 31‐fold increase in melatonin content compared to standard Williams 82, without detrimental impact on yield. Protein content was elevated, oil content reduced and the soybeans were suitable for post‐harvest processing. Furthermore, plants enriched in endogenous melatonin exhibited stronger resilience to adversity, evidenced by improved salinity tolerance in soybean seeds and increased resistance to *Verticillium dahliae* in cotton. Our research paves the way for the synthesis of target compounds in staple crops using synthetic genetic circuits, facilitating the development of novel biofortified crops to increase nutritional availability and environmental adaptability in the upcoming new era of agriculture.

## Introduction

Melatonin (N‐acetyl‐5‐methoxytryptamine) is a natural compound found across all living organisms and having diverse biological activities, including crucial roles in regulating circadian rhythms, ameliorating diabetes and mitigating inflammatory damage (Borjigin *et al*., 1995; Guo *et al*., 2021; Rusanova *et al*., 2019; Stein *et al*., 2020). In sleep regulation, melatonin modulates the timing of sleep onset and the sleep/wake cycle through its receptor‐dependent action on the suprachiasmatic nucleus (Kim *et al*., 2024). Modern lifestyle factors, such as increased stress, digital device addiction and irregular eating patterns, have exacerbated insomnia, driving rising global demand for melatonin supplements, particularly in developed nations (Rui, 2019). The market is projected to grow at an annual rate of 10% until 2027, primarily fuelled by increasing consumption in developing countries (Arnao *et al*., 2023).

Initially, melatonin was primarily extracted from animal pineal glands and urine, which posed a risk of viral transmission (Bonilla *et al*., 2004; Kennaway, 1993). With improvements in chemical synthesis methods, synthetic melatonin gradually replaced animal‐derived sources and became the dominant form used in industrial and medical applications (Hugel and Kennaway, 1995). Nevertheless, chemical synthesis generates persistent ecological contamination and undesirable by‐products, potentially resulting in serious health issues (Williamson *et al*., 1997, 1998). As such, exploring more sustainable and environmentally friendly biosynthetic pathways is essential. Plants, as synthetic biology platforms, can utilize carbon fixed through photosynthesis to synthesize target compounds, and they also contain other beneficial compounds for biological health, all while maintaining relatively low ecological footprints and production costs (Burnett and Burnett, 2020; Golubova *et al*., 2024). As consumers increasingly prioritize the transparency of intake sources and ingredients, plant‐based melatonin both provides a safer alternative and aligns with the principles of sustainable development (Arnao *et al*., 2023; Arnao and Hernández‐Ruiz, 2018).

Notably, melatonin also participates in a plethora of physiological processes within plants themselves. It was first identified in plants in 1995 (Dubbels *et al*., 1995; Hattori *et al*., 1995; Kolar *et al*., 1995) and has been characterized as a stimulator that promotes seed germination, root development and plant growth, as well as contributing to numerous adversity responses (Altaf *et al*., 2022; Arnao *et al*., 2022; Byeon and Back, 2014). Melatonin is especially prominent in relation to plant stress tolerance, with its levels increasing robustly to protect against biotic and abiotic stress (Raza *et al*., 2022).

A synthetic genetic circuit is a set of genetic devices, such as Boolean logic gates, toggle switches and feedback loops, designed with the guidance of mathematical models in a rational manner to construct a tunable and programmable genetic system (Hasty *et al*., 2002; Nielsen *et al*., 2016). The key to a standard and modular genetic logic gate lies in regulating pathway performance based on transcription factors, which bind with diverse cis‐regulatory elements (CREs) to establish transcription capacity and accurately regulate gene expression (Jiang *et al*., 2023; Jores *et al*., 2021). Synthetic genetic circuits can overcome the limitations of conventional native promoters with respect to transcription efficiency and strength, and unnecessary negative feedback can be avoided due to their orthogonality (Liu *et al*., 2013; Liu and Stewart, 2016).

The BUFFER gate is a commonly used genetic circuit that, within its framework, utilizes varying copy numbers of CREs as the input and produces different expression levels of a target product as the output (Lloyd *et al*., 2022). This approach enables higher expression intensity than natural promoters, with a broader dynamic range, while minimizing interference with other genes and pathways, thereby enhancing the likelihood of producing the desired traits (Liu and Stewart, 2016). To date, precisely defined genetic circuits have been produced in bacterial, yeast and animal systems (Gao *et al*., 2018; Schaumberg *et al*., 2016), but implementation in plants lags behind due to their less well‐understood genetic metabolism, complex systems and long life cycle. Inspiringly, a previous study reported the successful reprogramming of plant roots by synthetic genetic circuits in *Arabidopsis thaliana*, which represents the first attempt at tunable practice in stable plants (Brophy *et al*., 2022).

**Figure 1:**
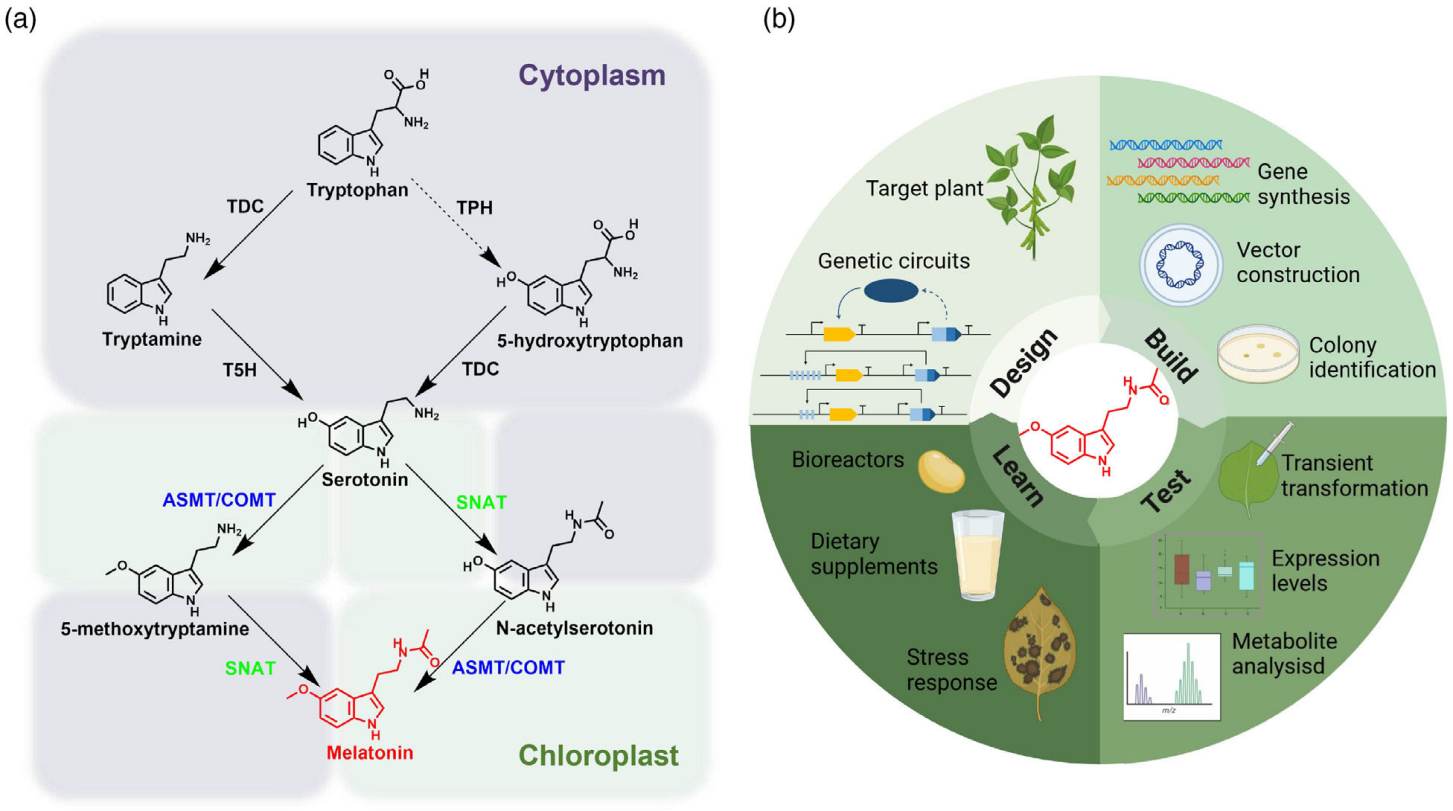
Melatonin biosynthesis in plants via genetic circuits. (a) The pathway of melatonin biosynthesis in plants includes key enzymes such as TDC, TPH (has not yet been identified in plants), SNAT, ASMT and COMT. Purple backgrounds indicate processes occurring in the cytoplasm, while green backgrounds indicate activities in chloroplasts. Dashed lines represent unconfirmed reactions. (b) Overview of the research process in this study, including Design‐Build‐Test‐Learn steps, which align with the principles of synthetic biology.

Melatonin in plants is synthesized from tryptophan through four enzymatic steps (Back *et al*., 2016). Initially, tryptophan decarboxylase (TDC) and tryptophan‐5‐hydroxylase (T5H) catalyse the conversion of tryptophan into serotonin. Serotonin is subsequently transformed into melatonin via the actions of serotonin N‐acetyltransferase (SNAT) and N‐acetylserotonin O‐methyltransferase (ASMT)/caffeic acid O‐methyltransferase (COMT) (Figure 1a). Furthermore, an alternative biosynthetic pathway has been proposed, in which tryptophan is first converted into serotonin through the combined actions of putative tryptophan hydroxylase (TPH) and TDC, although TPH has not yet been identified in plants (Back *et al*., 2016). While the specific biosynthetic pathways and organelles may vary across plant species, COMT has been demonstrated to be a key rate‐limiting enzyme in melatonin production (Byeon *et al*., 2014a).

Here, we explored the possibility of using synthetic genetic circuits to efficiently synthesize and accumulate melatonin in plants (Figure 1b). We first validated the transient expression of various BUFFER genetic circuit vectors in *Nicotiana benthamiana*, demonstrating the feasibility of this strategy for melatonin production. Subsequent implementation in soybean surprisingly revealed crop seeds to have enormous potential as platforms or plant factories for synthesizing and accumulating melatonin, achieving as much as 31‐fold greater content. Finally, we demonstrated that plants rich in endogenous melatonin possess enhanced resistance to adversity. Our work stands as an example of how new synthesis technologies can be applied to sustainably breed food crops that have customized composition and are economically climate resilient to diverse natural environments.

## Results

### Constructing BUFFER genetic circuits to synthesize melatonin in *N. benthamiana* leaves

**Figure 2:**
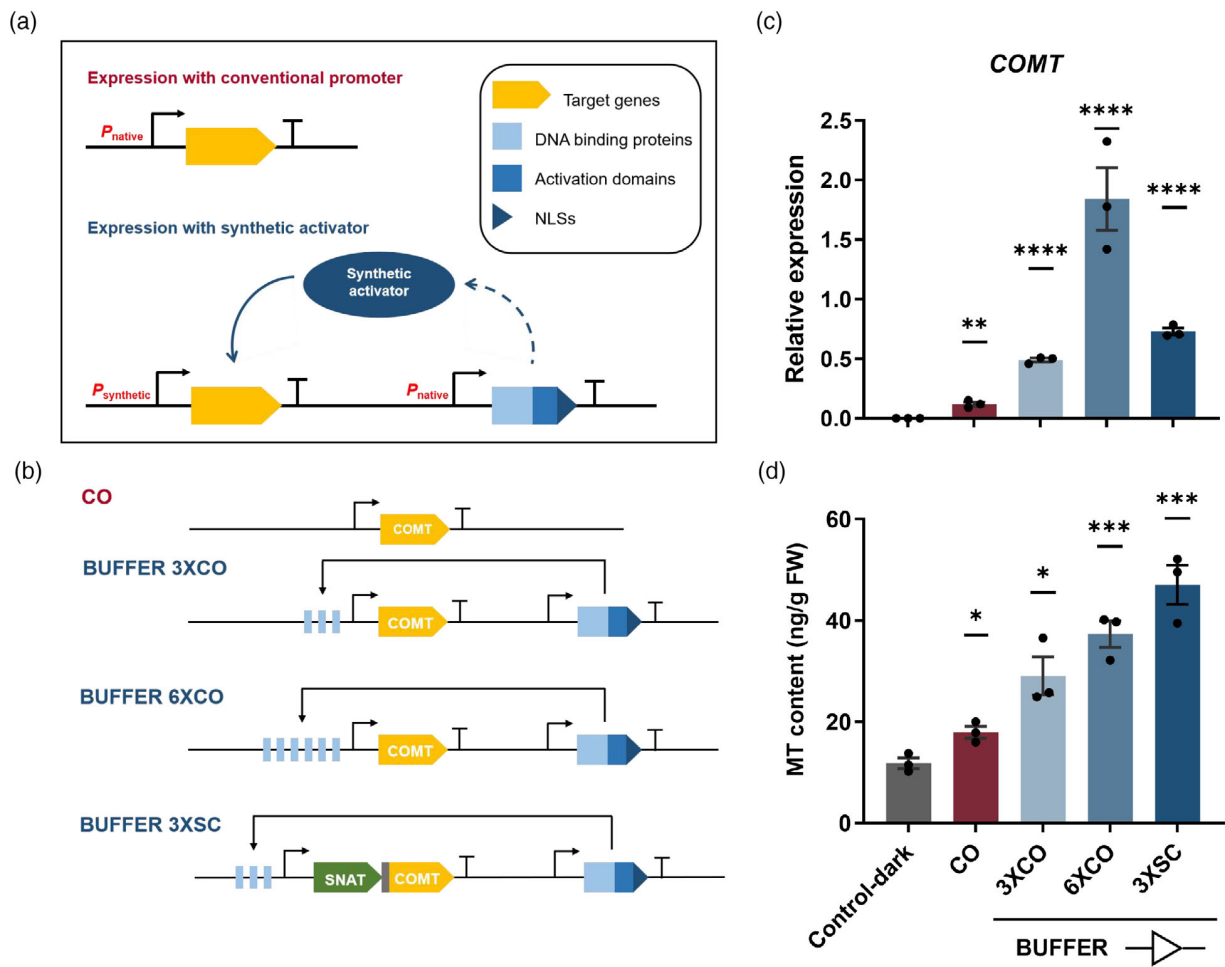
Reconstruction of synthetic genetic circuits in *N. benthamiana* leaves. (a) Schematic diagram of target gene expression driven by a conventional promoter and a synthetic activator. (b) Core components of the three synthesized BUFFER gates, 3XCO, 6XCO and 3XSC. The black curved arrow shows the transcription start site of the constitutive promoter, and the black ‘T’ indicates the terminator. (c) Expression of *COMT*, determined by qPCR. (d) Melatonin content in transient‐transformed leaves, detected by a reagent kit. FW, fresh weight. Data sourced from four to six leaves are presented as one biologically independent sample, expressed as mean values ± SEM, *n* = 3 biological replicates. Statistical significance of differences between control‐dark and transfected leaves was assessed using two‐tailed Student's *t*‐test (*P ≤ 0.05, **P ≤ 0.01, ***P ≤ 0.001, ****P ≤ 0.0001).

Unlike conventional promoters that directly drive the expression of target genes, synthetic activators drive expression through a set of synthetic transcriptional regulators (Figure 2a). To construct synthetic genetic circuits in plants, we first generated a set of synthetic transcriptional regulators composed of AmtR‐DNA‐binding proteins, ERF2 activation domains and SV40 nuclear localization signals (NLSs) (Brophy *et al*., 2022; Li *et al*., 2013). Notably, compared to the commonly used human herpesvirus‐derived VP16 activation domain, ERF2 activation domains derived from *Arabidopsis* exhibited greater effectivity in enhancing plant control over expression (Data S1), thereby making circuit design strategies more practical. In addition, we generated codon‐optimized versions of the pivotal plant melatonin biosynthesis genes *COMT* and *SNAT* (Data S2), based on the protein sequences of *Gossypium hirsutum* COMT and *Chlamydomonas reinhardtii* SNAT, respectively.

Next, using these synthetic regulators and target genes, we constructed circuits that perform BUFFER Boolean logic operations. The BUFFER genetic circuits can modulate the expression intensity of synthetic activators based on the number of binding sites, thereby accurately and controllably influencing the expression of target genes. The first two constructs, designated 3XCO and 6XCO, were created by fusing three and six expression‐activating binding sites, respectively. We chose three and six copies of the operator because a previous study indicated two copies to produce an expression level comparable to that of the G1090 promoter, while more copies resulted in higher expression (Brophy *et al*., 2022). To explore the impact of different target genes on compound content, we designed the third construct 3XSC, which included *COMT* and *SNAT* separated by a sequence encoding the 2A self‐cleavage peptide (Sharma *et al*., 2012). Additionally, we constructed a vector driven by the conventional CaMV 35S promoter (designated CO) to verify the facilitating effect of the synthetic activators on target compound synthesis (Figure 2b).

Subsequently, the constructed vectors were agroinfiltrated into *N. benthamiana* leaves using *Agrobacterium* strain GV3101 (p19). Four days post‐infiltration, expression in the leaves was analysed by quantitative real‐time PCR (qPCR), which confirmed the successful heterologous expression of *COMT*. As shown in Figure 2c, expression of *COMT* was significantly increased for the various BUFFER constructs compared to both the control‐dark and the conventional construct CO. Furthermore, as expected, 6XCO generated more transcripts than 3XCO, exceeding the normal expression level by a factor of three, and expression with 3XSC was slightly higher than 3XCO; the constructs therefore achieve precise control of *COMT* expression.

To further investigate the effects of the constructed promoters on melatonin content, we applied a detection kit to agroinfiltrated *N. benthamiana* leaves infected with the various constructs (Figure 2d, Figure S1). To our surprise, promoter 3XSC achieved the highest melatonin content among the three BUFFER genetic circuits. In addition, the melatonin content with 6XCO did not reach several fold higher than that produced with 3XCO, presumably due to insufficient precursor. Nonetheless, this set of BUFFER gates designed to express melatonin in *N. benthamiana* leaves demonstrates the reliability of genetic circuits and the feasibility of quantitative control of target compound content in plants.

### Seed‐specific expression enhances melatonin content in soybean

**Figure 3:**
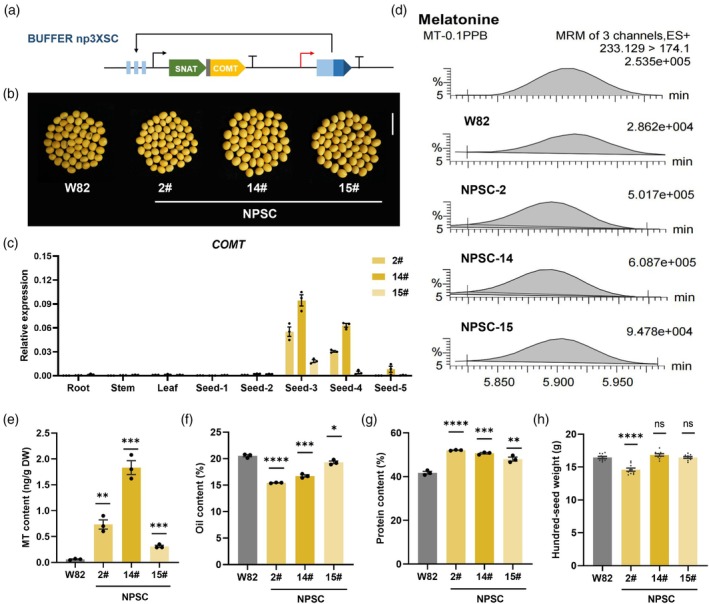
Seed‐specific expression enhances melatonin content in soybean. (a) Schematic diagram illustrating core components of the synthesized BUFFER gate for soybean transformation, np3XSC (NPSC). The red curved arrow indicates the transcription start site of the seed‐specific promoter. (b) T2 soybean seeds harvested from transgenic lines specifically expressing melatonin. Scale bar, 1.5 cm. (c) Expression of *COMT* in multiple tissues of transgenic soybeans. Seed 1 to Seed 5 indicate different stages at 3, 5, 6, 8 and 10 weeks post‐fertilization. (d) Melatonin detection based on a mass transition from *m/z* 233.1 to *m/z* 174.1, with a retention time of 5.90 min. (e) Melatonin content of soybean seeds, detected by UPLC‐MS/MS. (f, g) Oil and protein contents of transgenic lines compared to W82. (h) 100 ‐seed weight of transgenic lines compared to W82. DW = dry weight. Data are presented as mean values ± SEM, *n* = 3 biological replicates (c–g), and *n* = 10 biological replicates (h). Statistical significance of differences between W82 and transgenic lines was assessed using two‐tailed Student's *t*‐test (ns, no significance, *P ≤ 0.05, **P ≤ 0.01, ***P ≤ 0.001, ****P ≤ 0.0001).

Next, we explored whether our approach of melatonin biosynthesis using synthetic genetic circuits could be feasible and predictable as a stable genetic transformation in crops, which has been considered a promising biofortification platform to synthesize target compounds. Based on the melatonin contents obtained in transient transformation, we chose the highest yielding construct 3XSC as the core module for use in crops. To utilize this construct with plant seeds as the bioreactors, we developed another version (designated np3XSC, abbreviated as NPSC; Figure 3a) driven by a seed‐specific napin promoter, products downstream of which accumulate intensively in seeds (Ruiz‐Lopez *et al*., 2014). This construct was used for *Agrobacterium*‐mediated transformation to generate transgenic soybeans in the Williams 82 (W82) background (Figure 3b). PCR analysis confirmed the presence and stable inheritance of the transgenes in the T2 generation. We quantified the expression levels of the target gene across various tissues and observed that it is predominantly expressed in the seeds (Figure 3c, Figure S2). Subsequently, we used UPLC‐MS/MS to quantitatively detect the melatonin content of soybean seeds (Figure 3d). As shown in Figure 3e, all transgenic lines exhibited increased melatonin content compared with control W82 seeds. Surprisingly, one NPSC (14#) transgenic line achieved significantly elevated levels of melatonin, reaching 1.833 ng/g dry seed weight, a 31‐fold increase over the 0.059 ng/g dry seed weight in W82 seeds.

We additionally measured the oil and protein contents and observed a significant increase in protein levels in the transgenic lines, accompanied by a reduction in oil content (Figure 3f,g). We next examined major yield traits through a field trial, which revealed no statistically significant differences between NPSC and W82 plants (Figure 3h, Figure S3). Taken together, the above results suggest that seed‐specific expression utilizing synthetic genetic circuits enhanced the melatonin content in seeds without concomitant adverse effects in plants.

### Post‐harvest processing and metabolic profiling of biofortified soybean

**Figure 4:**
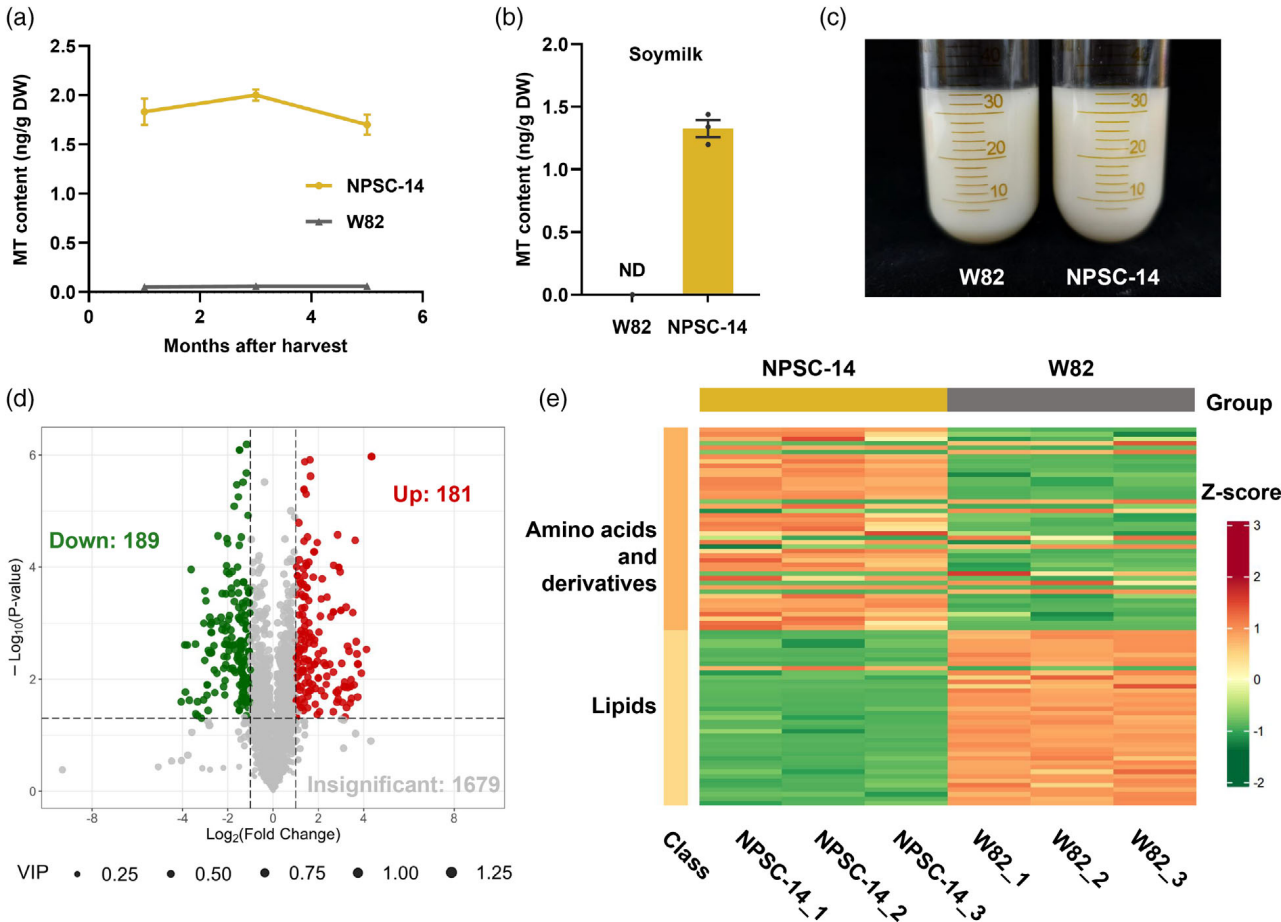
Post‐harvest processing and metabolic profiling of biofortified soybean. (a) Melatonin content in NPSC‐14 soybeans at 1, 3 and 5 months after harvest. (b) Melatonin content of soy milk, detected by UPLC‐MS/MS. (c) Appearance of soy milk. (d) Volcano plot of metabolites in NPSC‐14 and W82 seeds stored at room temperature for 3 months. Metabolites were identified and quantified using UPLC‐MS/MS. Each point represents a metabolite: green for downregulated, red for upregulated and grey for not significantly different. (e) Heat map of amino acids and derivatives and lipid metabolite pools. ND, not detected. Data are presented as mean values ± SEM, *n* = 3 biological replicates.

Post‐harvest processing, including both storage and cooking methods, is a critical determinant in the efficient utilization of biofortified crops (Huey *et al*., 2023). Accordingly, we assessed the impact of storage duration on our modified soybean, measuring the melatonin content of seeds stored at room temperature for 1, 3 and 5 months after harvest. Considering its superior melatonin content and absence of undesirable agronomic traits, we selected NPSC (14#) as the primary line for subsequent studies. The results indicated that, over the 5‐month storage period, melatonin content exhibited slight fluctuations but remained at elevated levels, confirming its stability in the modified soybeans after harvest (Figure 4a).

Soy milk, a traditional and highly favoured soy‐based product in Asia, has been an essential component of diet and culture for thousands of years (Tan *et al*., 2016). To investigate whether melatonin can be retained in soy milk for use as a dietary supplement, we produced soy milk from seeds of the NPSC (14#) transgenic line and the W82 control. Following grinding, boiling and filtering of residues, quantification determined the melatonin content in the clarified soy milk from NPSC (14#) seeds as 1.327 ng/g, whereas it was undetectable in W82 (Figure 4b,c). Relative to the melatonin content in the raw seeds, the soy milk preparation process achieved a utilization rate of 72.4%. This finding highlights the practical potential of biofortified soybeans.

After confirming the consistent increase of melatonin content in soybean seeds, we investigated the impact on other metabolites through non‐targeted UPLC‐MS/MS analysis. The volcano plot in Figure 4d illustrates the relative differences in metabolite abundance between NPSC (14#) and W82, along with their statistical significance. The corresponding heat map of differential metabolites is provided in Figure S4. Notably, NPSC (14#) exhibits a significant overaccumulation of amino acids and derivatives, along with a reduction of lipids (Figure 4e). These results align with the determined oil and protein contents. Additionally, greater levels of precursor substances involved in melatonin synthesis were observed in NPSC (14#) compared to W82 (Figure S5), indicating substantial accumulation across the entire biosynthetic pathway.

### Biofortified soybean seeds have improved tolerance of salinity during germination

Salt stress is a major abiotic factor that limits crop productivity and distribution, with salinized soil area increasing by 1.0–2.0 million hectares annually (Munns and Tester, 2008). To thrive in saline environments, plants must successfully complete seed germination, the initiation of their life cycle. Previous studies have demonstrated that exogenous melatonin can enhance salt tolerance in plants (Colombage *et al*., 2023). Thus, we sought to determine whether soybeans biofortified with endogenous melatonin possess superior salt tolerance in seed germination, conducting a comparative examination of NPSC (14#) and W82.

**Figure 5:**
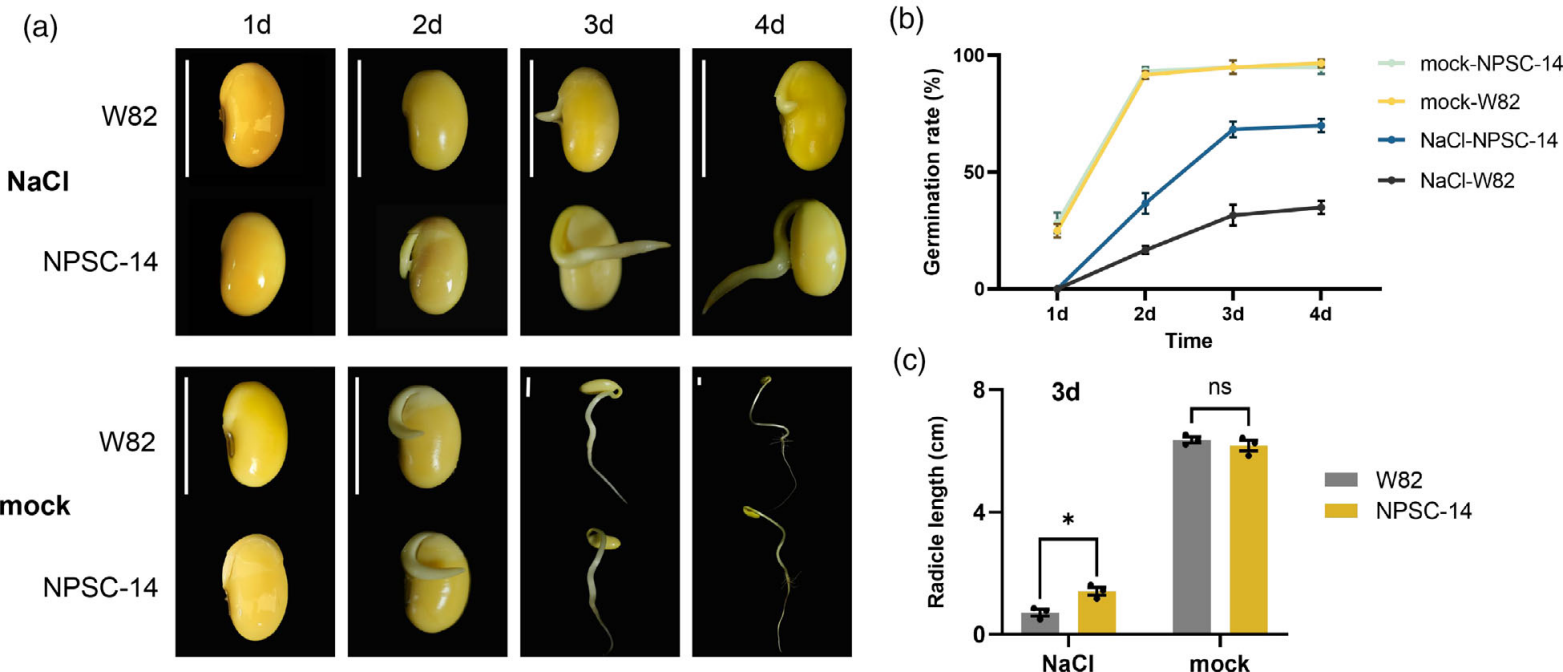
Biofortified soybean has improved salinity tolerance during seed germination. (a) Morphology of germinating seeds of NPSC‐14 and W82 under treatment with 150 mM NaCl or water (mock) for 4 days. Scale bar, 1 cm. (b) Germination rate of NPSC‐14 and W82 treated with 150 mM NaCl or mock, recorded daily. (c) Radicle length measured on day 3 of germination. Data sourced from 20 seeds are presented as one biologically independent sample, expressed as mean values ± SEM, *n* = 3 biological replicates. Significance of differences between treatments and controls was determined by multiple two‐way ANOVA (ns, no significance, *P ≤ 0.05).

Consistent with previous reports, salt treatment reduced both germination rate and the speed of germination (Figure 5a,b, Figure S6). After 2 days of 150 mM NaCl treatment, W82 and NPSC (14#) exhibited germination rates of 16.7% and 36.7%, respectively, while rates under the mock treatment exceeded 90%. By day 3, NPSC (14#) exhibited a higher germination rate (68.3%) than W82 (31.7%), with the rate plateauing by day 4. Salt stress also significantly impacted radicle length, with NPSC (14#) exhibiting a notable difference compared to W82 on day 3 (Figure 5c). These results demonstrate the melatonin biofortification of soybeans to enhance salt tolerance during seed germination, potentially expanding the area in which soybeans can be cultivated.

### Endogenous melatonin increases cotton resistance to *Verticillium dahliae*

**Figure 6:**
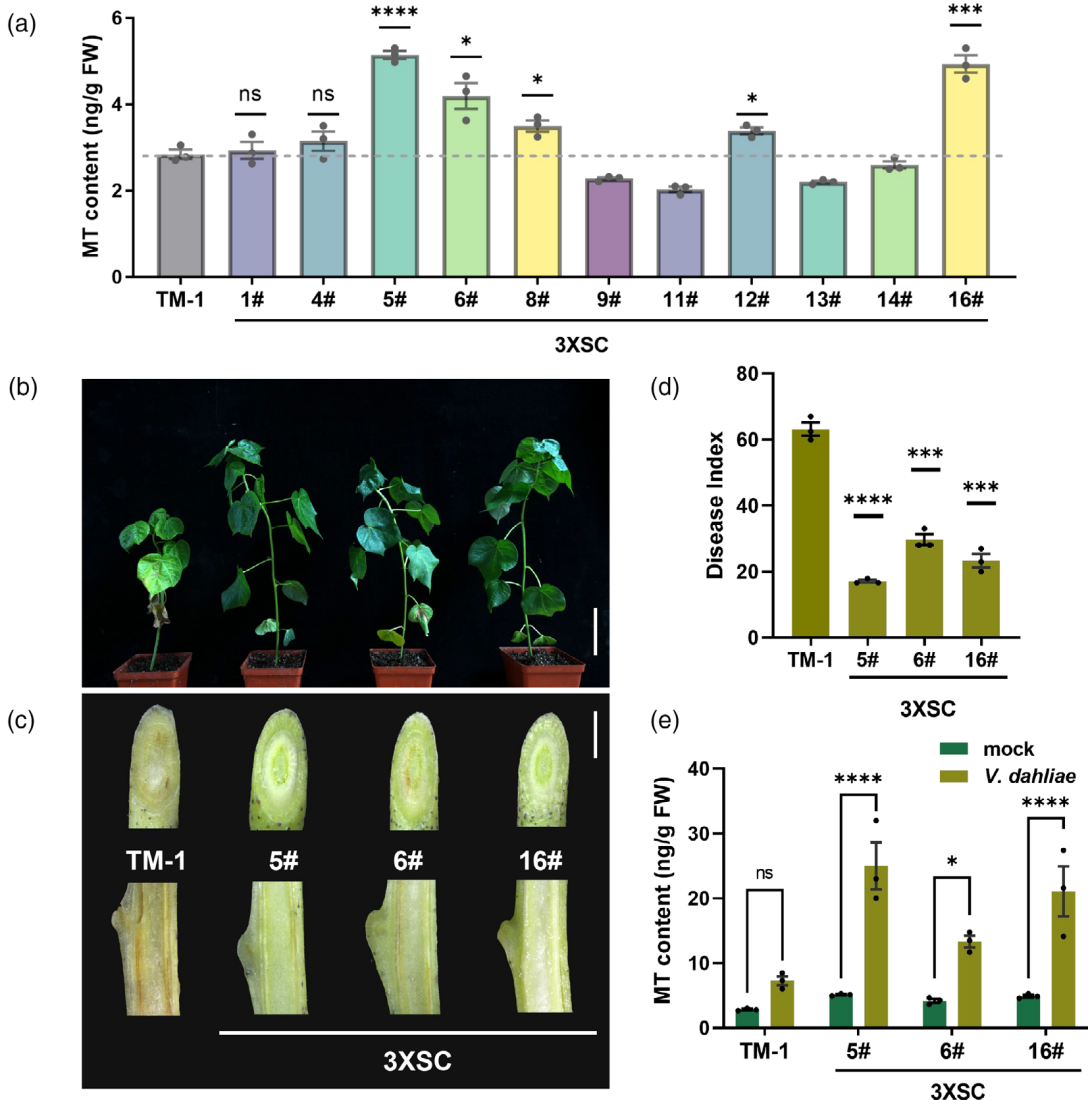
Endogenous melatonin increases cotton resistance to *V. dahliae*. (a) Melatonin content in leaves of genetically modified cotton lines under normal growth conditions. (b) Phenotypes of TM‐1 and transgenic plants at 20 days after *V. dahliae* inoculation. Scale bar, 8 cm. (c) Visualization by microscopy of oblique and longitudinal sections from brown discolorations on stems. Scale bar, 5 mm. (d) Disease index of TM‐1 and transgenic lines. FW = fresh weight. Data sourced from 4 to 6 (a) and 10 (d) plants are presented as one biologically independent sample, expressed as mean values ± SEM, *n* = 3 biological replicates. Statistical significance of differences between TM‐1 and transgenic lines was assessed using two‐tailed Student's *t*‐test (***P ≤ 0.001, ****P ≤ 0.0001). (e) Melatonin content in leaves under mock and *V. dahliae* infection conditions. Data sourced from four to six plants are presented as one biologically independent sample, expressed as mean values ± SEM, *n* = 3 biological replicates. Significance of differences was determined by multiple two‐way ANOVA (ns, no significance, *P ≤ 0.05, ****P ≤ 0.0001).

The soil‐borne pathogen *V. dahliae* is a grievous concern in the cotton industry, as it causes a vascular disease that induces leaf defoliation and vascular tissue browning, ultimately leading to plant death (Qiu *et al*., 2023). We transferred the carrier 3XSC into *G. hirsutum* acc. TM‐1 and produced more than 10 transgenic lines. Under natural growth conditions, plants of overexpressing lines were shorter than TM‐1 due to shortened internodes, but demonstrated normal flowering and fruiting. Determinations of melatonin content revealed leaves of the transgenic lines to have levels approximately 0.7‐ to 1.8‐fold greater than the WT (Figure 6a). We also observed increased melatonin levels in transgenic cotton seeds (Figure S7), highlighting the potential utility of such genetic modification for enhancing the use of cotton seeds as a novel oilseed crop.

Next, we inoculated three T1 transgenic lines with *V. dahliae*. At 20 days post infection (dpi), TM‐1 exhibited more chlorotic and wilted leaves compared to the transgenic lines (Figure 6b). Microscopic analysis revealed that transgenic stems had fewer lesion areas than those of TM‐1 plants (Figure 6c). In terms of disease symptoms, the transgenic lines displayed significantly lower disease indices (Figure 6d). Additionally, we measured melatonin content in the infected leaves at 10 dpi, with the transgenic lines again exhibiting significantly higher melatonin levels compared to TM‐1 (Figure 6e). These results indicated that overexpression of *COMT* and *SNAT* in cotton enhanced plant resistance to *V. dahliae*, suggesting a valid role of synthetic genetic circuits.

## Discussion

### Soybean as an ideal crop for melatonin biofortification

Plant‐based chassis offers a promising and sustainable platform for synthetic production of natural dietary supplements. Unlike microbial systems that rely on sugar‐derived raw materials, photosynthetic plants offer a sustainable option (Matthews *et al*., 2019), in which existing metabolic precursors and cofactors are utilized for the synthesis of bioactive compounds such as melatonin (Patron, 2020). Notably, plants rich in melatonin also contain other compounds beneficial for biological health, including antioxidants and vitamins (Arnao *et al*., 2023); as such, they can provide a comprehensive and widely appealing nutritional supplement. Synthetic phytomelatonin thus holds significant potential in addressing the global market's growing interest in natural and functional foods.

In the face of global population growth and the limited availability of arable land, the pressure to enhance crop productivity and nutritional value has never been greater. Soybeans are a primary source of protein and other essential nutrients for human diets and the food industry worldwide (List, 2015). Notably, soybeans exhibit higher melatonin concentration compared to grains and corn (Sangsopha *et al*., 2020). Furthermore, legumes are typically rich in micronutrients and amino acids, often surpassing cereals, which makes them an excellent vehicle for biofortification (Rehman *et al*., 2019). Therefore, we focused on utilizing soybeans as an ideal chassis for synthetic melatonin production with the goal of meeting personalized nutrition aims while retaining the crop's fundamental properties.

### Synthetic genetic circuits for targeted metabolite production in plants

Recent advances in synthetic genetic circuits allow for the precise control of gene expression to obtain desired traits (Xia *et al*., 2019). In contrast to native promoters, which are regulated by complex endogenous transcription factors and may introduce unwanted variability (Liu *et al*., 2013), synthetic promoters are optimized for efficiency and precision. By excluding distant regulatory elements like enhancers and silencers, synthetic promoters minimize background noise and ensure tighter control over the engineered pathways. Hence, they offer a more streamlined and effective method for enhancing melatonin synthesis in plants.

However, modification of genetic circuits in plants is yet challenging due to the higher systemic complexity and longer research cycle in plant systems compared to prokaryotic systems. Current research primarily focuses on using GFP or LUC signals as outputs (Jores *et al*., 2021; Schaumberg *et al*., 2016), which has limited application in practical plant trait genetic engineering. In this study, we successfully constructed BUFFER genetic circuits centred on synthetic melatonin. In transient transformation, we observed that the actual concentrations produced across different BUFFER gates deviated from the linear ratio predicted by fluorescence‐based signals in previous studies, likely due to substrate limitations. Nevertheless, integrating multiple pathway genes into genetic circuits enhanced the overall yield of the target compounds. These findings provide valuable insights for future metabolic engineering of plants.

### Nutritional and environmental benefits of phytomelatonin‐enriched crops

Through seed‐specific expression in soybean, we achieved phytomelatonin levels of 1.833 ng/g DW, a 31‐fold increase over wild‐type W82 seeds (0.059 ng/g DW). In cotton, a constitutive promoter drove more moderate accumulation, from 0.38 ng/g to 0.70 ng/g DW in seeds, reflecting a broader but less concentrated distribution across tissues. These enhancements are meaningful in both nutritional and agronomic contexts, especially given the crops' retained yield performance. Meanwhile, non‐targeted metabolomics revealed a significant increase in protein content, alongside reduced oil content. These findings are consistent with previous studies suggesting that melatonin plays a role in inhibiting oil accumulation in seeds, potentially through its effects on lipid metabolism (Li *et al*., 2020). This metabolic shift may be particularly beneficial in protein‐focused applications, such as plant‐based meat or high‐protein animal feed systems.

Encouragingly, our findings indicate the potential of melatonin‐enriched soybeans to be retained during storage and processing. We successfully detected melatonin in soy milk derived from biofortified soybeans after 3 months of storage at room temperature, a feature not found in standard soybeans. This supports the feasibility of using melatonin‐enriched crops in functional foods, responding to consumer interest in convenient, health‐promoting dietary options.

In addition to nutritional benefits, melatonin‐enriched plants also exhibit enhanced resistance to environmental stresses. As global climate change and land degradation intensify, the area of saline‐alkali land continues to expand, particularly in China, where it has reached 99 million hectares, severely affecting agricultural productivity (Chen *et al*., 2024). Our study shows that biofortified soybeans exhibit enhanced salt tolerance, which is consistent with previous findings that seed coating with melatonin can improve salt tolerance in soybeans (Wei *et al*., 2015), providing a promising solution for cultivation on saline‐alkali soils.

In parallel with our work on biofortified soybeans, we have also focused on enhancing melatonin levels in cotton, a vital global agricultural crop. Existing studies have shown that melatonin treatment enhances cotton's tolerance to *V. dahliae* by increasing lignin and gossypol accumulation with increased melatonin levels (Li, He *et al*., 2019; Zeng *et al*., 2022). Our research further supports these findings, showing that elevating endogenous melatonin levels enhances cotton's resistance to *V. dahliae*, which holds promise for practical applications in cotton disease‐resistant breeding.

Plant melatonin serves various functions beyond salt tolerance and *V. dahliae* resistance. Exogenous applications, through seed priming, foliar spraying or root treatment, can improve stress tolerance and delay senescence in plants (Arnao and Hernández‐Ruiz, 2019; Jan *et al*., 2023). For example, rice treated with 100 μM melatonin under cold stress showed a 1.4‐fold increase in chlorophyll and a 33% reduction in H₂O₂ (Han *et al*., 2017). Genetic strategies, such as overexpression of melatonin biosynthetic genes like *TDC3*, can further elevate melatonin levels (Byeon *et al*., 2014b). Moreover, phytomelatonin has benefits for animal health, as demonstrated by improved sperm quality in rams fed a phytomelatonin‐enriched diet (Peña‐Delgado *et al*., 2023).

### Commercialization potential and practical applications

Most commercial phytomelatonin supplements are derived from plant extracts. Extracts from *Tanacetum parthenium* and *Hypericum perforatum* have been developed into supplements delivering up to 5 mg of nearly pure phytomelatonin per capsule (Murch *et al*., 1997; Murch and Saxena, 2006). Some medicinal plants can accumulate 80–100 mg/kg DW of phytomelatonin, especially when elicited (Pérez‐Llamas *et al*., 2020). However, this method is species‐specific, involves costly purification and limits scalability. In contrast, biofortified soybeans can integrate melatonin production into existing agricultural systems, enabling direct dietary intake and simplified processing.

While chemical melatonin remains inexpensive, with 95% purity available at approximately EUR 0.25–0.3 per gram (Arnao *et al*., 2023), its production generates environmental and safety concerns. In contrast, melatonin derived from plants offers a natural, sustainable alternative that aligns with consumer demand for health‐conscious products (Cano *et al*., 2024). Rather than replacing synthetic forms, phytomelatonin complements them as a cleaner source to improve well‐being. Melatonin‐enriched crops also offer agro‐economic benefits, including enhanced stress tolerance, added nutritional value and greater income stability for farmers in marginal areas. While initial costs and regulatory testing are significant, they are largely upfront and amortizable and can be offset by the scalable use of existing agricultural systems (Nestel *et al*., 2006). Broader adoption will depend on cost‐effective production, supportive policies and public acceptance (Sandhu *et al*., 2023).

The use of genetically modified organisms (GMOs) offers both benefits and challenges. They can enhance crop nutritional content, as seen with Golden Rice and omega‐3 fish oil crops, addressing hidden hunger and providing necessary health supplements (Napier *et al*., 2015; Paine *et al*., 2005). Additionally, GMOs can increase plant resilience to environmental stresses, reduce disease‐induced crop losses and decrease reliance on chemical pesticides, contributing to more sustainable agriculture (Giudice *et al*., 2021; Van Esse *et al*., 2020). However, challenges remain. Intellectual property, regulatory approval, public engagement and social consent must be carefully addressed to ensure a balance between innovation, sustainability and safety (Napier *et al*., 2019).

Overall, this study not only provides a new approach for the plant‐based synthesis of melatonin but also offers strong support for addressing the growing demand for health‐conscious products and advancing sustainable agricultural practices. Looking ahead, we anticipate that, after overcoming challenges related to production costs and market acceptance, melatonin‐enriched crops will be widely applied in personalized nutrition. At the same time, this research offers valuable insights into the potential of using plant chassis for the synthesis of novel functional foods and nutritional supplements, fostering synergistic development of agricultural production and human health as well as creating a more sustainable and health‐oriented food system.

## Materials and methods

### Plant materials and growth conditions

*Nicotiana benthamiana* was cultivated in a growth chamber at 20 °C–22 °C, under a photoperiod of 16 h light and 8‐h darkness. Genetic transformation experiments utilized the soybean cultivar W82 (*G. max*) and the cotton cultivar TM‐1 (*G. hirsutum*). Soybean and cotton plants were grown in pots within a greenhouse maintained at 28 °C with a 16‐h light and 8‐h dark cycle and 60% relative humidity. Yield evaluations of biofortified and wild‐type soybeans were completed in a field in Hangzhou (Zhejiang, China), ensuring consistent planting conditions. Fresh leaves from these plants were harvested for genomic DNA and total RNA extraction. All plant materials were promptly frozen in liquid nitrogen and subsequently stored at −70 °C.

### Construction of vectors

The transcriptional activator elements (including various copies of the operator, binding proteins, activation domains and NLSs) and codon‐optimized target genes (*COMT, SNAT*) used in this study were synthesized by GenScript (Nanjing). Specifically, these constructs incorporated the synthetic activator AmtR‐ERF2‐NLS driven by a constitutive G1090 promoter and terminated by E9, along with the target gene *COMT*, which was regulated by multiple copies of the operator bound by AmtR to a minimal plant promoter (CaMV 35S promoter) and terminated by ADH. These four constructs were subsequently subcloned into the pCambia2300 vector. Using homologous recombination with ABclonal 2X MultiF Seamless Assembly Mix (RK21020), the sequences were assembled to create a set of BUFFER gates. The sequences utilized in this study can be found in the Data S1.

### Transient expression in *N. benthamiana*

Various vectors' pending validation was transformed into *Agrobacterium tumefaciens* strain GV3101 (p19), which contains the P19 silencing suppressor. Transient expression in 5‐week‐old *N. benthamiana* leaves was carried out as previously described (Zheng *et al*., 2021). Briefly, *Agrobacterium* cultures were transferred into LB medium and incubated overnight at 220 rpm and 28 °C to an OD600 of 0.8–1.0. Next, cells were collected by centrifugation and washed with infection buffer (10 mM MgCl_2_, 10 mM MES, 150 μM acetosyringone, pH 5.7). After 4 days of agro‐infiltration, leaves were harvested, frozen in liquid nitrogen and stored at −80 °C until metabolite extraction and measurement.

### Stable transformation of soybean and cotton

Stable genetic transformation of W82 by *Agrobacterium tumefaciens* strain EHA105 was accomplished via electroporation. Soybean seeds germinated for 1 day served as the source material, and explants were subjected to infection by placing them at room temperature for 30 min. Co‐cultivation was carried out in the dark at 23 °C for 3 days, after which explants were transferred to a resting medium. Following 7 days at 25 °C, the explants were placed on an appropriate selective medium for 3 weeks to induce resistant shoots. The shoots were then transferred to an elongation medium with the appropriate selective pressure and cultured under light for 6–9 weeks. Finally, the elongating regenerated seedlings were subjected to rooting to produce transgenic plants. The method for cotton genetic transformation mediated by *A. tumefaciens* strain GV3101 followed a previous description (Li, Wang *et al*., 2019).

### Quantitative real‐time PCR

Total RNA from the various samples was extracted with the EASYspin Plus Plant RNA Kit (Molfarming, RK16‐50T) and was reverse‐transcribed to cDNA using the HiScript II Reverse Transcriptase Kit (Vazyme, R201‐01) according to the manufacturer's instructions. Quantitative real‐time PCR was performed on StepOne & StepOnePlus Real‐Time PCR Systems (Thermo Fisher Scientific) following the instructions of the ChamQ Universal SYBR qPCR Master Mix Kit (Vazyme, Q711‐02). The reference genes Nt*EF1a* (GenBank accession no. AF120093), Gm*Actin11* (GenBank accession no. AK285830) and Gh*His3* (GenBank accession no. AF024716) were used for normalization of target gene expression. Primers used for qPCR are listed in Table S1. The E^−ΔΔCt^ method was used to calculate gene relative expression. All experiments were conducted with three biological replicates and three technical replicates.

### Determination of melatonin content using UPLC‐MS/MS

Melatonin quantification in soybean seeds and soy milk was carried out using UPLC‐MS/MS, a highly sensitive, specific and precise analytical technique (Wang *et al*., 2021). Dried soybean seeds from three independent biological replicates were pulverized into a powder, and their analysis was conducted using the Waters Acquity Premier Xevo TQ Absolute platform. In brief, 500 mg of soybean powder was extracted with 3.0 mL of 80% aqueous methanol by ultrasonication for 20 min at 4 °C. Subsequently, the mixture was incubated in the dark at 4 °C for 12 h. After centrifugation at 2000 rpm and 4 °C for 20 min, the supernatant was transferred to a new tube. The remaining residue was washed with 1 mL of 80% aqueous methanol and combined with the supernatant for analysis. The analytical conditions were as follows: samples were separated using an Atlantis BEH C18 column (100 × 2.1 mm, 1.7 μm) with a column temperature of 35 °C. The flow rate was set at 0.3 mL/min. The mobile phases were 0.1% formic acid (A) and acetonitrile (B), and the gradient was as follows: 100% A for 8 min; 65%–35% A for 5 min; 100% B for 4 min. Mass spectrometry was performed in Multiple Reaction Monitoring mode. The source parameters in positive polarity were set as follows: Capillary voltage: 3 kV; desolvation gas temperature: 500 °C; desolvation gas flow rate: 1000 L/h; cone gas flow rate: 150 L/h; and ion source temperature: 150 °C.

### Determination of melatonin content using ELISA

An ELISA kit (CBMORE, CA01089P‐96T) was employed to rapidly and efficiently quantify melatonin content in *N. benthamiana* and *G. hirsutum* leaves. Fresh samples were ground in liquid nitrogen and then mixed with physiological saline according to the manufacturer's instructions. All experiments were conducted in six replicates.

### Non‐targeted metabolomics analysis with UPLC‐MS/MS

Biological samples were subjected to vacuum freeze‐drying using a lyophilizer. The dried samples were then ground into fine powder utilizing a grinder at a frequency of 30 Hz for 1.5 min. Subsequently, 50 mg of the powdered sample was accurately weighed with an electronic balance and mixed with 1200 μL of a pre‐cooled (at −20 °C) 70% methanolic aqueous internal standard extract. The mixture was vortexed for 30 s every 30 min, repeated a total of six times. Afterwards, the samples underwent centrifugation at 12 000 rpm for 3 min, and the supernatant was collected. The resulting solution was filtered through a microporous membrane with a pore size of 0.22 μm and stored in an injection vial for subsequent UPLC‐MS/MS analysis. In the differential metabolite analysis of W82 and NPSC‐14, the selection criteria consisted of a VIP threshold (VIP > 1) and an absolute log_2_ fold change threshold (|Log_2_FC| ≥ 1.0). VIP values were extracted from the OPLS‐DA results. Prior to OPLS‐DA, the data were subjected to log transformation (log_2_) and mean centring. To prevent overfitting, a permutation test was performed with 200 permutations. The specific upregulated and downregulated differential metabolites are shown in Tables S2 and S3.

### Determination of oil and protein contents

For oil content determination, dried samples from three independent biological replicates were ground into a powder, which was then placed in a Soxhlet extractor and continuous reflux extraction with petroleum ether was performed for 3 h. After extraction, the samples were dried at 100 °C for 1 h, cooled and weighed. For protein content determination, dried samples from three independent biological replicates were digested and subsequently placed in a Kjeldahl nitrogen analyser for automatic nitrogen detection. The nitrogen was ultimately absorbed in a boric acid solution in a receiving flask, followed by titration and calculation. All experiments were conducted in three replicates.

### Germination test

Twenty seeds of W82 and NPSC‐14, sourced from the same year and field, were sterilized by soaking in a 5% sodium hypochlorite solution for 15 min, followed by three rinses with deionized water. The seeds were then placed in 9 cm diameter Petri dishes lined with filter paper. The salt treatment group was exposed to a 150 mM NaCl solution, while the mock group was treated with distilled water. To minimize evaporation, the Petri dishes were covered and incubated in the dark at 25 °C. Germination was considered to occur when the radicle length reached 2 mm. Germination progress was recorded every 24 h under dark conditions, with the medium replaced at each interval. Germination characteristics were assessed based on the germination rate and radicle length; that is, germination rate = number of germinated seeds/total number of seeds and radicle length = ∑ length of germinated seeds/number of germinated seeds. The experiment was repeated three times.

### Infection of cotton by *V. dahliae* and disease assessment

Mycelia of Vd991, a virulent defoliating *V. dahliae* strain, were initially grown on potato dextrose agar medium, then collected and cultured in liquid Czapek's medium at 25 °C under oscillation (150 rpm) for 4 days. One‐month‐old cotton seedlings were inoculated with Vd991 (1 × 10^7^ conidia/mL), with control seedlings receiving water addition instead. Then, plants were cultured in a growth chamber at 25 °C under a 16‐h light/8‐h dark cycle. After 20 days, the disease state was scored as previously described (Zhou *et al*., 2021): 0, healthy, no wilting, yellowing or dropping‐off; 1, one or two cotyledons with symptoms; 2, a single true leaf with symptoms; 3, more than two leaves with symptoms; 4, death, all true leaves dropped off. The disease index (DI) was calculated from observations of 10 individual plants with the following formula: DI = [(∑disease grades × number of infected plants)/(total checked plants × 4)] × 100. Data are means ± SEM of three biological replicates.

### Statistical analysis

The data provided in this study are expressed as mean ± SEM from at least three biological replications unless otherwise specified. A two‐tailed paired Student's *t‐*test was used to assess significant differences between two samples. Statistical analyses were performed using GraphPad Prism v.9.

## Conflict of interest

The authors declare no conflict of interest.

## Author contributions

T.Z. conceived the research project and designed the experiments. Y.S. constructed the genetic circuits in soybean and cotton. Z.H. analysed the non‐targeted metabolomics data. Y.S., X.M. and Z.S. contributed to evaluate the tolerance of soybean seeds to salt. L.H., W.Z., J.Z. and Y.S. performed the cotton *V. dahliae* resistance experiment. T.Z., Y.S., Z.H. and Y.H. participated in writing and revising the manuscript. All authors discussed results and commented on the manuscript.

## Supporting information


**Figure S1** Standard curve of melatonin content, determined by UPLC‐MS/MS (a) and ELISA kit (b).
**Figure S2** Expression of *SNAT* in various tissues of biofortified soybeans.
**Figure S3** Field evaluation of major yield traits in biofortified soybeans.
**Figure S4** Heat map of differential metabolites between NPSC‐14 and W82 seeds.
**Figure S5** Relative abundance of precursor metabolites in the melatonin biosynthesis pathway.
**Figure S6** Phenotypic germination of soybean seeds under salt and mock treatments.
**Figure S7** Phenotype of modified cotton seeds (a) and melatonin content as detected by UPLC‐MS/MS (b).
**Table S1** Primers used for RT‐qPCR of target genes.
**Table S2** List of upregulated differential metabolites.
**Table S3** List of downregulated differential metabolites.
**Data S1** Sequences of synthetic transcriptional activator elements.
**Data S2** Codon‐optimized sequences.

## Data Availability

The data that supports the findings of this study are available in the supplementary material of this article.
