# Supplementary material for: Enhancing melatonin biosynthesis in crops through synthetic genetic circuits: A strategy for nutritional fortification in soybean and stress resistance in cotton
Source: Plant Biotechnol J. 2025 Jul 7;23(10):4428–39. doi: 10.1111/pbi.70253 (PMC12483955; doi:10.1111/pbi.70253)
Supplement: Supplementary file 1 — Figure S1 Standard curve of melatonin content, determined by UPLC‐MS/MS (a) and ELISA kit (b). Figure S2 Expression of SNAT in various tissues of biofortified soybeans. Figure S3 Field evaluation of major yield traits in biofortified soybeans. Figure S4 Heat map of differential metabolites between NPSC‐14 and W82 seeds. Figure S5 Relative abundance of precursor metabolites in the melatonin biosynthesis pathway. Figure S6 Phenotypic germination of soybean seeds under salt and mock treatments. Figure S7 Phenotype of modified cotton seeds (a) and melatonin content as detected by UPLC‐MS/MS (b). Table S1 Primers used for RT‐qPCR of target genes. Table S2 List of upregulated differential metabolites. Table S3 List of downregulated differential metabolites. Data S1 Sequences of synthetic transcriptional activator elements. Data S2 Codon‐optimized sequences. [file PBI-23-4428-s001.docx]

**Supporting Information**

**Figure S1**. Standard curve of melatonin content, determined by UPLC-MS/MS (a) and ELISA kit (b).

**Figure S2**. Expression of *SNAT* in various tissues of biofortified soybeans.

**Figure S3**. Field evaluation of major yield traits in biofortified soybeans.

**Figure S4**. Heatmap of differential metabolites between NPSC-14 and W82 seeds.

**Figure S5**. Relative abundance of precursor metabolites in the melatonin biosynthesis pathway.

**Figure S6**. Phenotypic germination of soybean seeds under salt and mock treatments.

**Figure S7**. Phenotype of modified cotton seeds (a) and melatonin content as detected by UPLC-MS/MS (b).

**Table S1**. Primers used for RT-qPCR of target genes.

**Table S2**. List of upregulated differential metabolites.

**Table S3**. List of downregulated differential metabolites.

**Data S1**. Sequences of synthetic transcriptional activator elements.

**Data S2**. Codon optimized sequences.


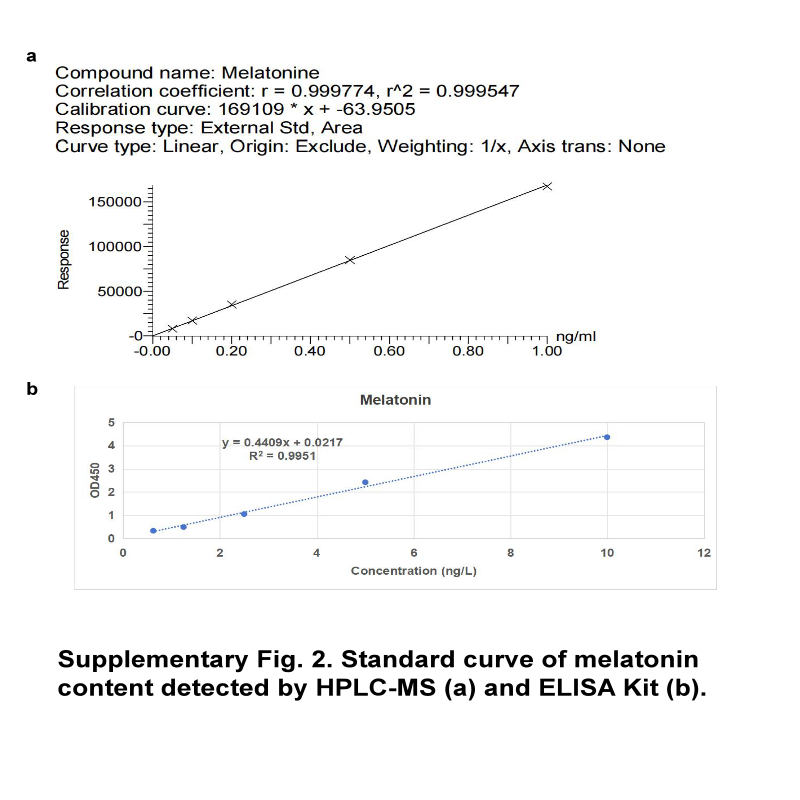


**Figure S1. Standard curve of melatonin content, determined by UPLC-MS/MS (a) and ELISA kit (b).**

**
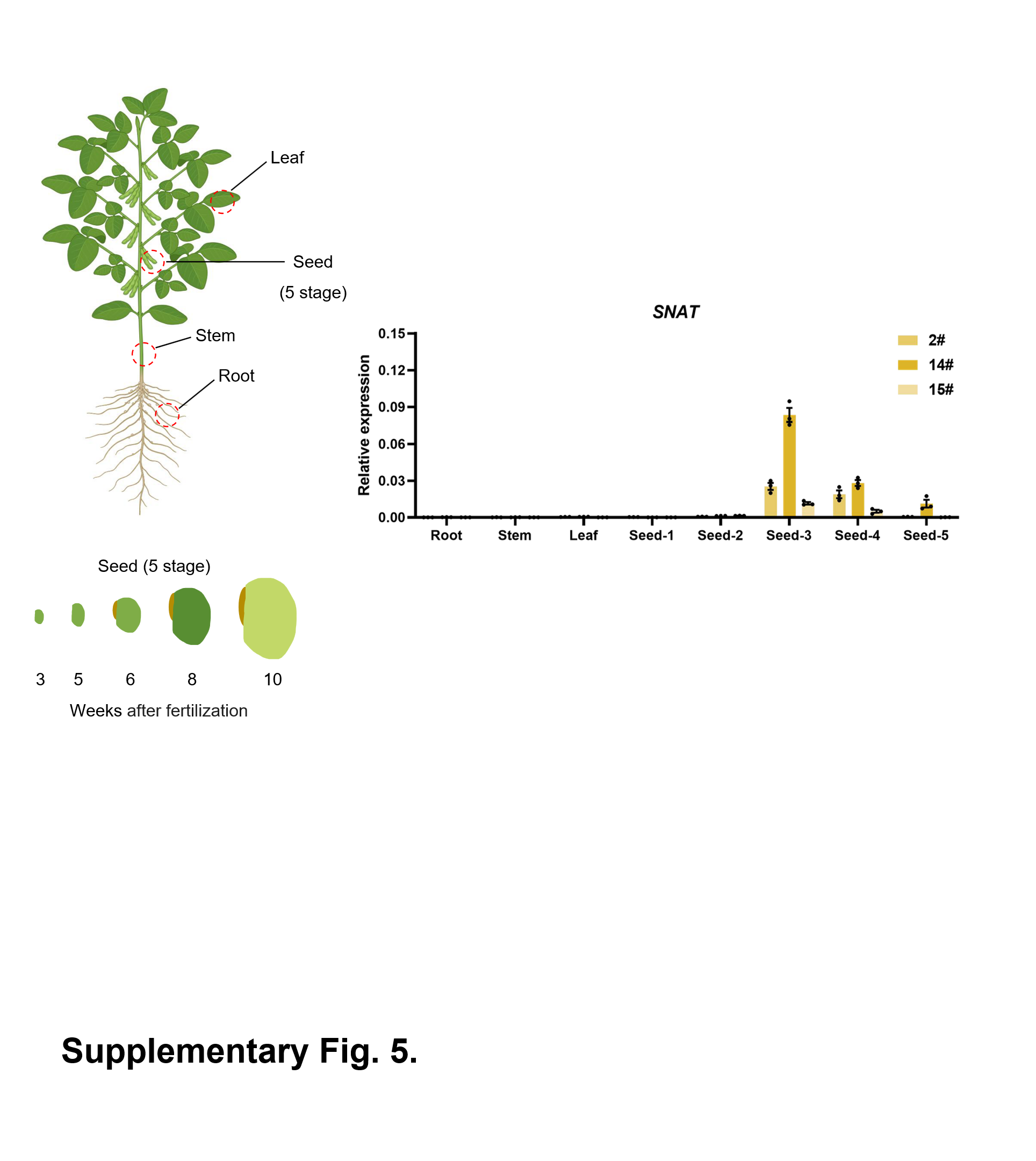
**

**Figure S2. Expression of *SNAT* in various tissues of biofortified soybeans.**

**
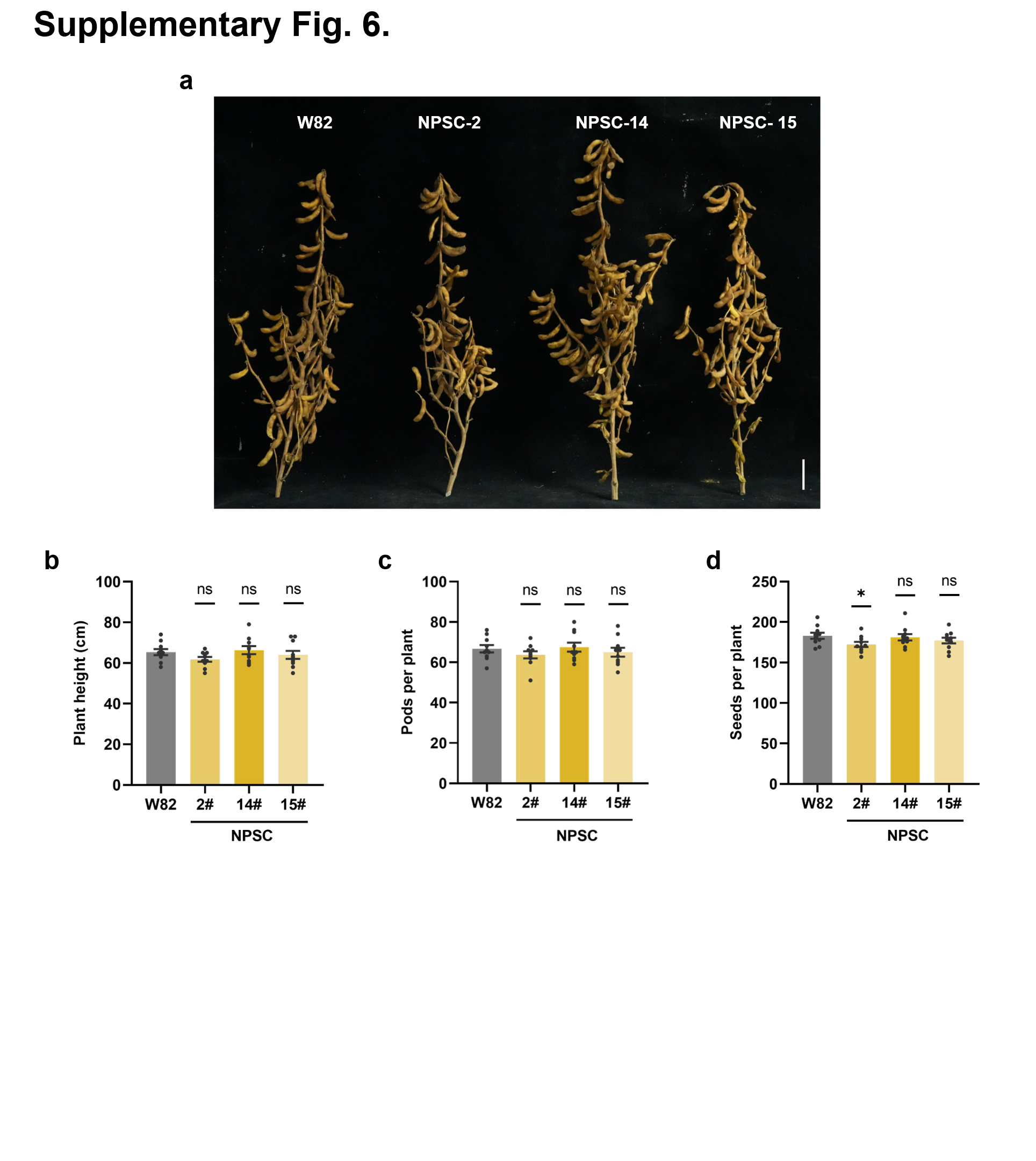
**

**Figure S3. Field evaluation of major yield traits in biofortified soybeans.**

**
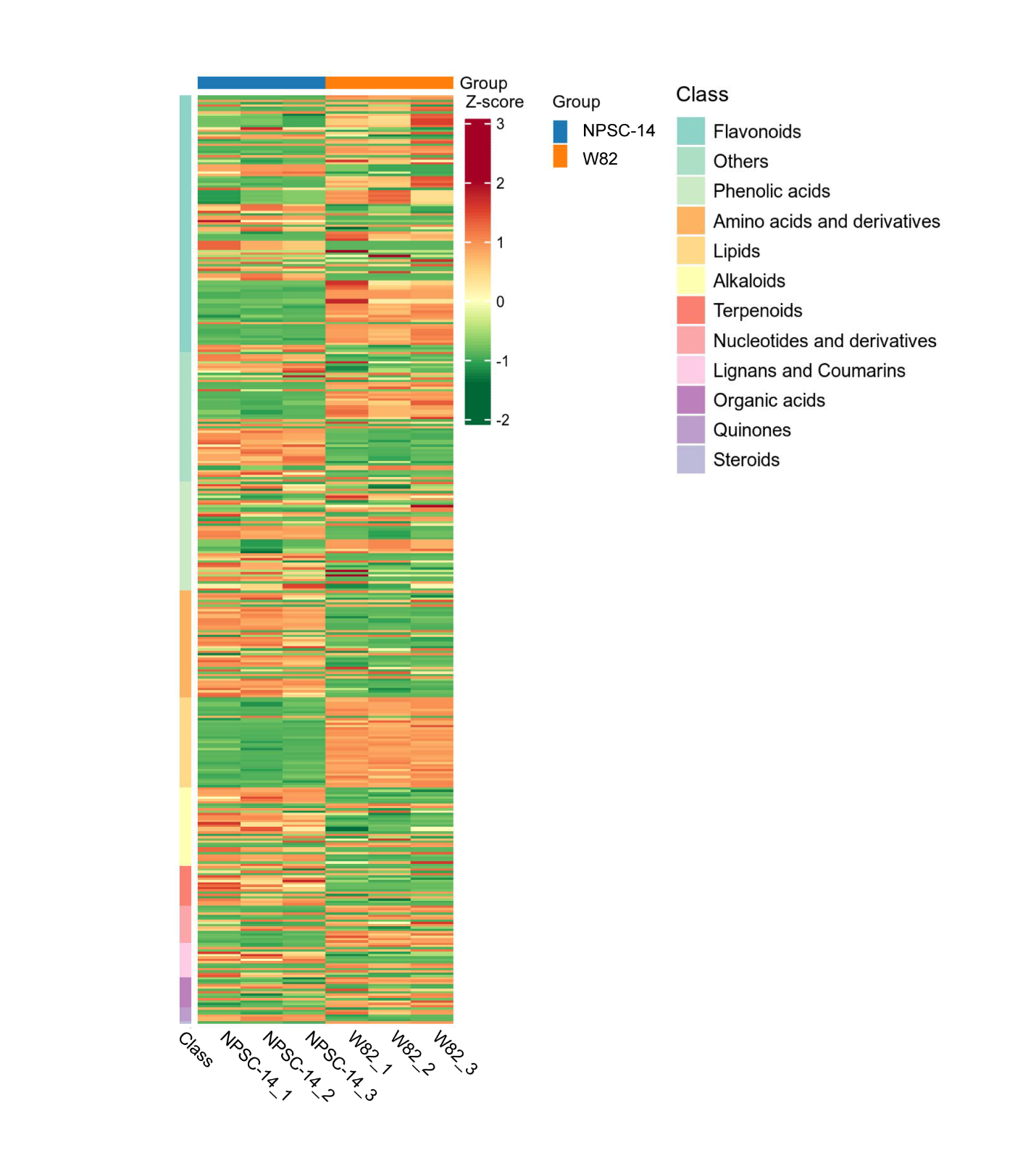
**

**Figure S4. Heatmap of differential metabolites between NPSC-14 and W82 seeds.**


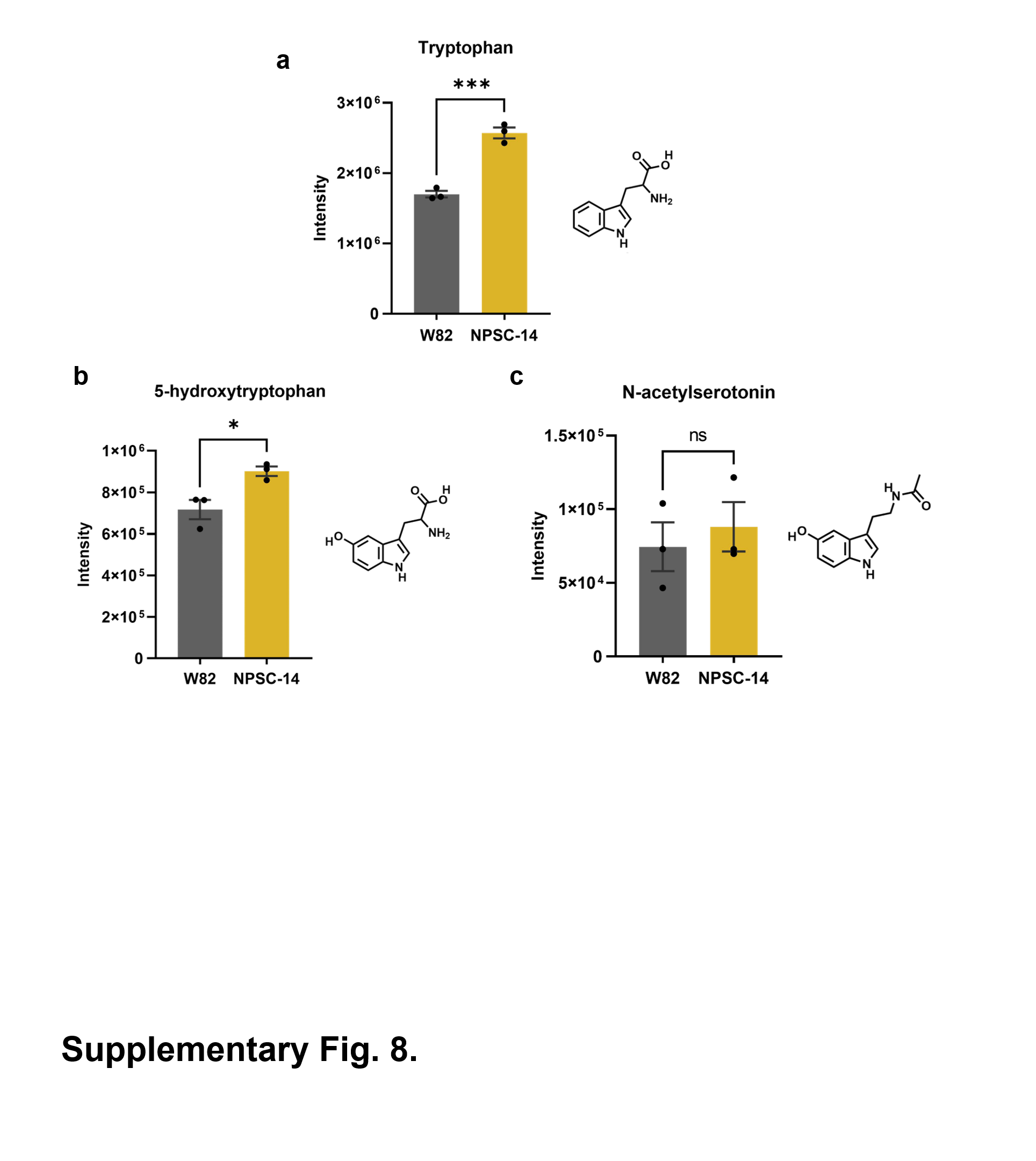


**Figure S5. Relative abundance of precursor metabolites in the melatonin biosynthesis pathway.**

**
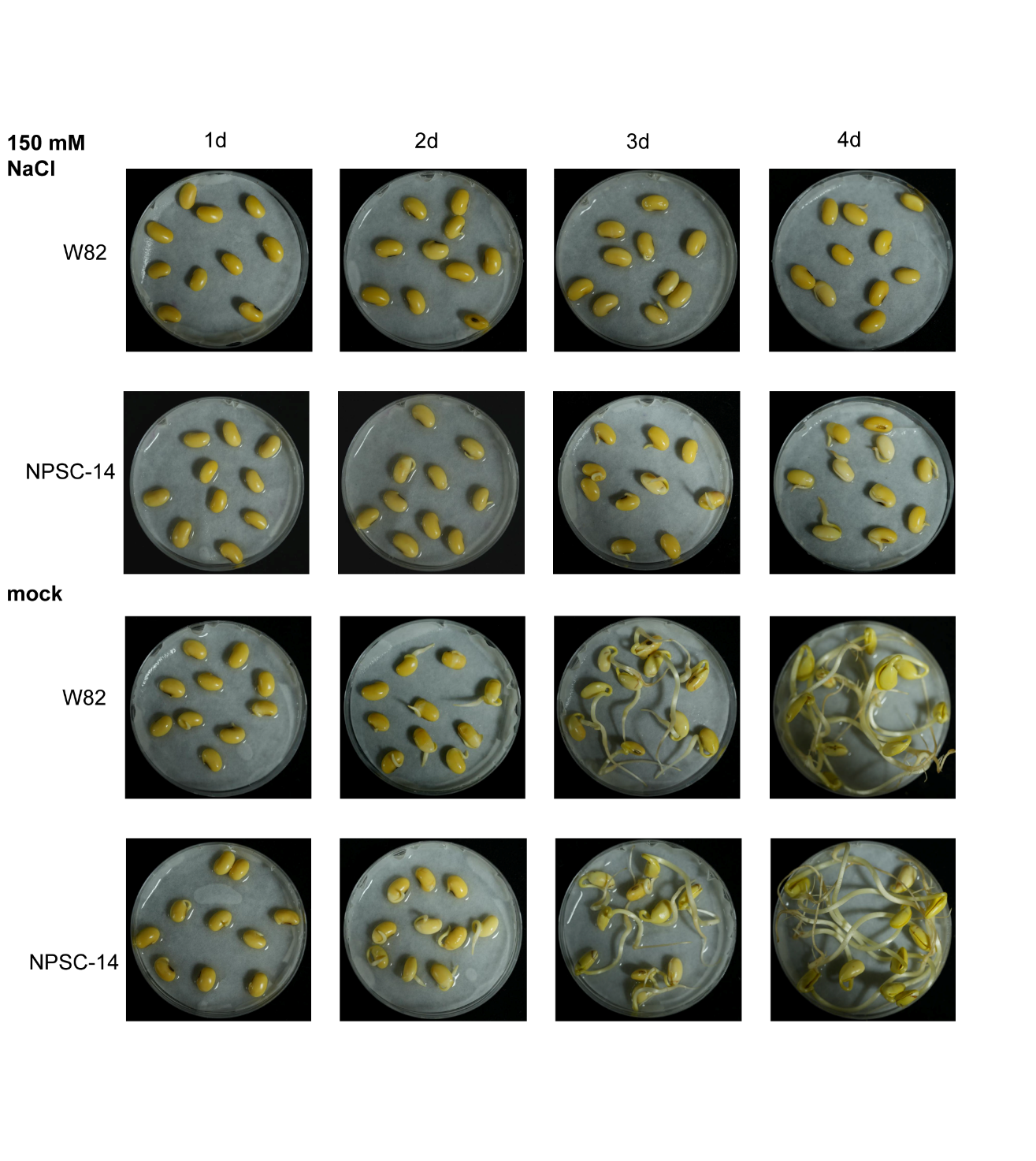
**

**Figure S6. Phenotypic germination of soybean seeds under salt and mock treatments.**

**
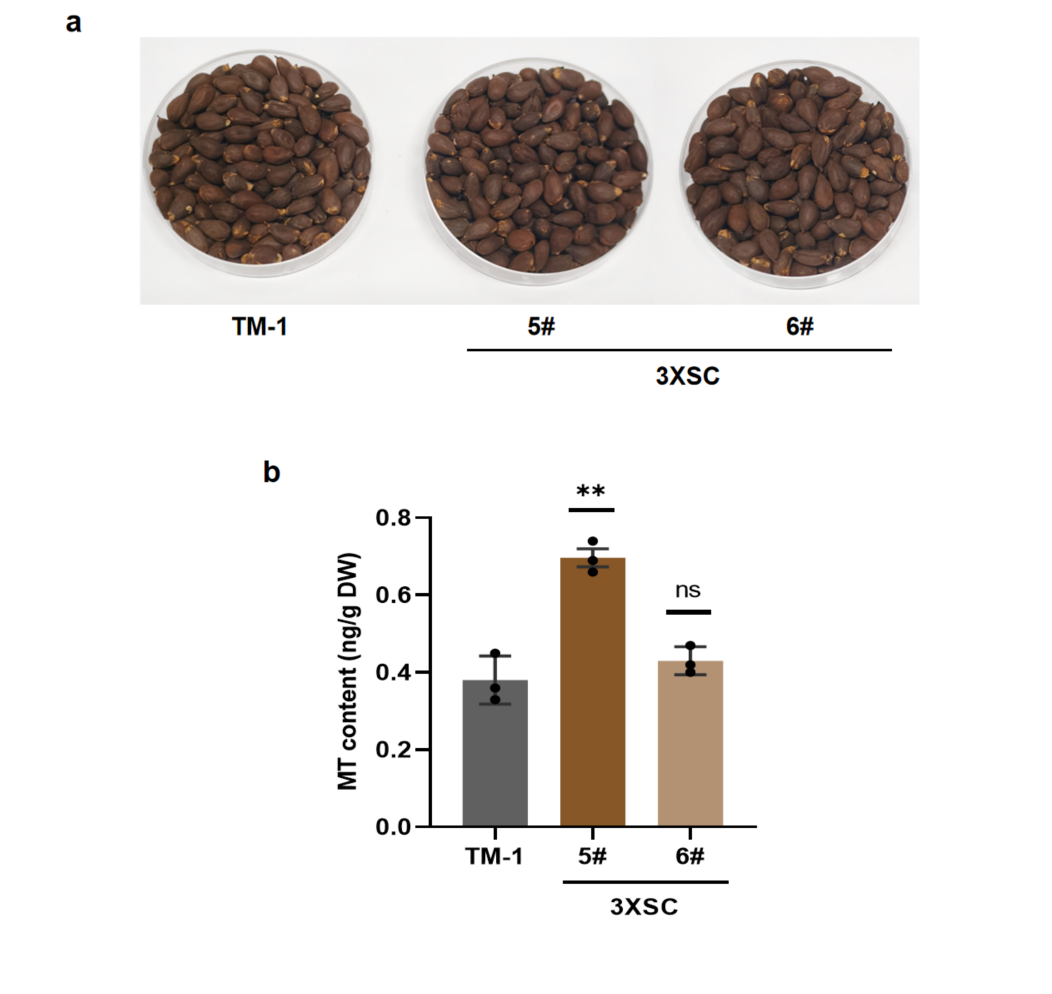
**

**Figure S7. Phenotype of modified cotton seeds (a) and melatonin content as detected by UPLC-MS/MS (b).**

**Table S1. Primers used for RT-qPCR of target genes.**

| Name | Primer (5'-3') |
| --- | --- |
| EF1a_F | TGAGATGCACCACGAAGCTC |
| EF1a_R | CCAACATTGTCACCAGGAAGTG |
| Actin11_F | ATGTAGCCATCCAGGCTGTT |
| Actin11_R | ACCAGCCAGATCAAGACGAA |
| His3_F | CGGTGGTGTGAAGAAGCCTCAT |
| His3_R | AATTTCACGAACAAGCCTCTGGAA |
| COMT_F | TCATGCTGGACCGTATCTTG |
| COMT_R | AGAGTCTTTCCACTTTGCCA |
| SNAT_F | GATGTGGATGTCTACGAGCTTC |
| SNAT_R | GAACCAGGCAATTTCTTAACGG |

**Table S2. List of upregulated differential metabolites between NPSC-14 and W82 (the top 30).**

| **Compounds** | **Class I** | **Type** |
| --- | --- | --- |
| Leu-Leu-Phe | Amino acids and derivatives | up |
| portulacanone D glucoside | Flavonoids | up |
| Prolylproline | Amino acids and derivatives | up |
| Val-Val-Leu | Amino acids and derivatives | up |
| Acetamiprid | Alkaloids | up |
| 2,4,2',4'-tetrahydroxy-3'-prenylchalcone | Flavonoids | up |
| Genistein-7-O-(6''-malonyl)glucoside | Flavonoids | up |
| Gentianamine | Alkaloids | up |
| Apigenin-8-C-glucoside-7-O-Sophoroside | Flavonoids | up |
| Chrysoeriol-5,7-di-O-glucoside | Flavonoids | up |
| Anisic acid-O-feruloyl glucoside | Phenolic acids | up |
| Soyasaponin H glucuronic acid glucose rhamnoside | Terpenoids | up |
| 1-O-Feruloyl-beta-D-glucose | Phenolic acids | up |
| 3'-O-Methyl-epicatechin | Flavonoids | up |
| Sulfurein; Sulfuretin-6-O-glucoside | Flavonoids | up |
| Styrylamine | Alkaloids | up |
| 4,5,6-Trihydroxy-2-cyclohexen-1-ylideneacetonitrile | Alkaloids | up |
| 5-Hydroxyquinoline | Alkaloids | up |
| 2,6-Dimethoxy-1,4-benzoquinone* | Quinones | up |
| Cys-Gln | Amino acids and derivatives | up |
| ethyl-L-arginine* | Amino acids and derivatives | up |
| 4-Ethynylbenzaldehyde | Others | up |
| (S)-2-Phenyloxirane | Others | up |
| Octinoxate | Phenolic acids | up |
| Retronecine | Alkaloids | up |
| 3-(Pyrazol-1-yl)-L-alanine | Amino acids and derivatives | up |
| L-Phenylalanine | Amino acids and derivatives | up |
| 2-(2-Ethynyl-2,4-cyclopentadien-1-ylidene)ethenol | Others | up |
| Dalbergin malonyl glucoside | Lignans and Coumarins | up |
| Formononetin-7-O-(6''-Malonyl)glucoside | Flavonoids | up |

**Table S3. List of downregulated differential metabolites between NPSC-14 and W82 (the top 30).**

| **Compounds** | **Class I** | **Type** |
| --- | --- | --- |
| Naringenin chalcone; 2',4,4',6'-Tetrahydroxychalcone | Flavonoids | down |
| N-Acetyl-L-tyrosine | Amino acids and derivatives | down |
| 9R-hydroxy-10E,12Z-octadecadienoic acid | Lipids | down |
| 3',4',7-Trihydroxyflavone | Flavonoids | down |
| 1-Oleoyl-Sn-Glycerol | Lipids | down |
| Octadeca-9,12,15-trienoic acid* | Lipids | down |
| Gorlic acid* | Lipids | down |
| Punicic acid (9Z,11E,13Z-octadecatrienoic acid) | Lipids | down |
| 3,4-Dihydro-4-(4'-hydroxyphenyl)-5,7-dihydroxycoumarin glucoside | Lignans and Coumarins | down |
| Isosalipurposide (Phlorizin Chalcone) | Flavonoids | down |
| 3-Hydroxy-1,2-dimethoxyxanthone-glucoside | Others | down |
| 6,2'-Dihydroxyflavone | Flavonoids | down |
| Daidzein | Flavonoids | down |
| Chrysin-8-C-glucoside | Flavonoids | down |
| Naringenin-6-C-Glucoside | Flavonoids | down |
| p-Coumaric acid | Phenolic acids | down |
| N-Acetyl-L-leucine | Amino acids and derivatives | down |
| (+)-Peusedanol | Lignans and Coumarins | down |
| 5,6,7-Trihydroxy-8-methoxyflavone* | Flavonoids | down |
| Tectorigenin* | Flavonoids | down |
| Hispidulin (5,7,4'-Trihydroxy-6-methoxyflavone)* | Flavonoids | down |
| 6,7,8-Tetrahydroxy-5-methoxyflavone | Flavonoids | down |
| 9-Hexadecenoic acid | Lipids | down |
| 5,7,2'-Trihydroxy-8-methoxyflavone; Scutevulin* | Flavonoids | down |
| Estrane-3,17-diol | Steroids | down |
| Tadehaginoside | Phenolic acids | down |
| 3'-O-methylorobol* | Flavonoids | down |
| Succinyladenosine | Nucleotides and derivatives | down |
| 5-Aminovaleric acid | Organic acids | down |
| N-Methyl-L-proline* | Amino acids and derivatives | down |

**Data S1. Sequences of synthetic transcriptional activator elements**

>AmtR-ERF2-NLS

ATGGCTGGCGCCGTGGGCAGACCCAGAAGATCTGCTCCTCGGAGAGCCGGCAAGAACCCCCGGGAAGAGATTCTGGATGCCAGCGCCGAGCTGTTCACCAGACAGGGCTTTCACCACCAGCACCCACCAGATTGCCGACGCTGTGGGCAGACAGGCCAGCCTGTACTACCACTTCCCCAGCAAGACCGAGATCTTCCTGACCCTGCTGAAAAGCACCGTGGAACCCTCCACCGTGCTGGCCGAGGATCTGTCTACCCTGGACGCCGGACCCGAAATGAGACTGTGGGCTATCGTGGCCAGCGAAGTGCGGCTGCTGCTGAGCACCAAGTGGAACGTGGGCCGGCTGTACCAGCTGCCCATCGTGGGCTCTGAGGAATTCGCCGAGTACCACAGCCAGCGCGAGGCCATGACCAACGTGTTCAGAGATCTGGCCACCGAGATTGTGGGCGACGACCCCAGAGCCGAACTGCCCTTCCACATCACCATGAGCGTGATCGAGATGCGGCGGAACGACGGCAAGATCCCTAGCCCTCTGAGCGCCGACTCTCTGCCCGAGACAGCCATTATGCTGGCTGACGCCTCCCTGGCTGTGCTGGGAGCACCTCTGCCTGCCGACAGAGTGGAAAAGACACTGGAACTGATCAAGCAGGCCGACGCCAAGGGAGGAGGTGGATCAGAATCCGACTACGCTTTGTTGGAGTCGATAACACGTCACTTGCTAGGAGGAGGAGGAGAGAACGAGCTGCGACTCAATGAGTCAACACCGAGTTCGTGTTTCACAGAGAGTTGGGGAGGTTTGCCATTGAAAGAGAATGATTCAGAGGACATGTTGGTGTACGGACTCCTCAAAGATGCCTTCCATTTTGACACGTCATCATCGGACTTGAGCTGTCTTTTTGATTTTCCGGCGCCGAAGAAGAAGAGGAAGGTT

>AmtR binding operator

TTCTATCGATCTATAGATAAT

>Minimal 35S promoter

CACAATCCCACTATCCTTTCGCAAGACCCTTCCTCTATATAAGGAAGTTCATTTCATTTGGAGAGAACACGGGGGACTCTAGA

**Data S2. Codon optimized sequences**

>*COMT* gene, codon optimized

ATGGGTTCAATCGGTGAAACTCAAATGACACCCACCCAAGTCTCAGATGAGGAAGCCAACTTATTCGCTATGCAACTTGCCAGTGCATCAGTTCTCCCCATGGACCTCAAATCAGCCATTGACTTGACTTGCTGGAGATCATGGCCAAAGCTGGTCCAGGTGCTTTCTTGTCCCCAAAAGAAGTGGCTTCCAAGCTCCCCACCACCAACCCTGATGCACCCGTCATGCTGGACCGTATCTTGCGTCTCCTGGCTAGCTACAACGTCCTCACTTGCTCCTTGCGTACCCTTCCTGATGGCAAAGTGGAAAGACTCTATGGCCTTGGCCCTGTCTGCAAATTCTTGACCAAGAACGAAGATGGTGTCACTCTTTCCGCCCTTAGTCTCATGAATCAAGACAAGGTCCTTATGGAGAGCTGGTACTACTTGAAAGATGCTGTGCTGGATGGTGGAATTACATTCAACAAGGCCTATGGTATGACTGCATTTGAGTACCATGGCACTGATACTAGATTCAACAAGGTTTTCAACAGGGGAATGTCTGATCACTCTACCATCACCATGAAGAAGATTCTCGATACATATGATGGTTTCCAAGGACTAAAAACATTGGTCGATGTTGGCGGTGGTACCGGTGCCACGCTTAGCATGATCGTCTCTAAGTACCCCACCATAAAAGGCATTAACTTCGATTTGCCTCATGTCATTGAGGATGCTCCTAGCTGTCCTGGTGTGGAGCATGTTGGTGGAGACATGTTTGTAAGTGTACCAAAAGGAGATGCCATTTTCATGAAGTGGATATGTCATGATTGGAGCGACGAACACTGCGCCAAGTTTTTGAAGAACTGCTATGAAGCTTTGCCAGACAACGGGAAAGTGATTGTTGCCGAATGCATTCTTCCTGATTACCCCGAACCTAGCCTTGCCACGAAGTTGGTTGTCCATATTGATTGCATCATGTTGGCTCACAACCCTGGTGGGAAAGAGAGGACCGCAAAGGAATTCAAGGCACTAGCAACGGGTGCGGGATTTCAAGGCTTCCAAATCAAATGCTCCGCTTTTGGCACCAACATCATGGAGTTTCTCAAAAGTGTTTGA

>*SNAT* gene, codon optimized

ATGGCTGAAGAGTCTTTGGATGCTCAGTTCAACCTCTTGGTTCTACAGTTTTCTTTGGACCTGTTCAACCAGAGATGTTGGATAGGATTCATGAGCTTGAAGCTGCTTCATATCCAGAGGATGAAGCTGCTACTTACGAGAAGTTGAAATTCAGAATCGAAAATGCTTCTAACGTTTTTCTTGTTGCTTTGTCAGCTGAGGGTGATGAACCTAAGGTTGTTGGTTTCGTTTGTGGAACTCAAACAAGGGCTTCTAAACTTACACATGAGTCTATGTCAACCCATGATGCTGATGGTGCTCTTTTGTGCATTCATTCAGTTGTTGTTGATGCTGCTCTTAGAAGGAGAGGATTGGCTACTAGGATGCTTAGAGCTTATACAGCTTTCGTTGCTGCTACCTCTCCAGGTTTGACTGGAATCAGACTTTTGACCAAGCAAAACCTTATCCCTTTGTATGAAGGTGCTGGATTTACTCTTTTGGGTCCATCAGATGTTGAGCAAGCTGATTTGTGGTACGAATGTGCTATGGAGCTTGAAGCTGAAGAGGAAGCTGAGGCTGCTGAAGCTTAA
